# Supplementary material for: Phylogenetic Analysis Reveals Four New Species of Otidea from China
Source: Biology (Basel). 2022 Jun 6;11(6):866. doi: 10.3390/biology11060866 (PMC9219986; doi:10.3390/biology11060866)
Supplement: Supplementary file 1 [file biology-11-00866-s001.zip › Table S1.pdf]

Table S1. Information on sequences used in molecular phylogenetic analyses for *Otidea*.

Note: "—" shows no sequence in the GenBank database. GenBank accession numbers for sequences generated in this study are in boldface.

Species name	Specimen voucher	Locality	ITS	LSU	*ef1-α*	*rpb2*
Monascella botryosa	CBS 233.85	Spain	MH861870	MH873558	KC109256	JX943831
O. adorniae	MCVE 30102	Italy	MK850486	MK850502	—	—
O. adorniae	MCVE 30106	Italy	MK850484	MK850500	—	—
O. adorniae	MCVE 30105	Italy	MK850483	MK850499	—	—
O. alutacea	S-F257085	Italy	KM010069	KM823192	KM823260	KM823386
O. alutacea	FLAS-F59409	Pakistan	MN495935	MN493146	—	—
O. alutacea	KH.09.133	Norway	KM010071	KM823185	KM823253	KM823381
O. alutacea	ARAN A3023204	Spain	KM010072	KM823186	KM823254	KM823382
O. alutacea	S-F257084	Italy	KM010075	KM823464	—	—
O. alutacea	OSC 56777	USA	AF072071	AF086582	—	—
O. alutacea	OSC 56798	USA	AF072072	AF086583	—	—
O. alutacea	OSC 56747	USA	AF072070	KM823189	KM823257	—
O. alutacea	OSC 56770	USA	AF072073	AF086585	—	—
O. alutacea	KH.09.135	Norway	KM010064	KM823190	KM823258	KM823384
O. alutacea	KH.10.198	Sweden	KM010065	KM823459	—	—
O. alutacea	KH.13.50	Sweden	KM010076	KM823465	—	—
O. alutacea	JS.08.43	Sweden	KM010063	KM823458	—	—
O. alutacea	KH.09.178	Sweden	KM010066	KM823191	KM823259	KM823385
O. alutacea	K(M)142010	England	KT818924	—	—	—
O. alutacea	KS-94-192	Denmark	KM010067	KM823460	—	—
O. alutacea	Moorefun19	USA	KM010070	KM823194	KM823262	KM823387
O. alutacea	OSC 56782	USA	AF072076	AF086586	—	—
O. alutacea	OSC 56813	USA	AF072075	AF086584	—	—
O. alutacea	OSC 56758	USA	AF072074	KM823193	KM823261	—
O. alutacea	KH.07.46	Denmark	KM010061	KM823457	—	—
O. alutacea	C-F-48045	Sweden	KM010068	KM823461	—	—
O. alutacea	WZ 2123 (HMAS 72058)	China	KU987013	KU987025	—	—
O. alutacea	WZ 2128 (HMAS 72057)	China	KU987014	KU987026	—	OM792167
O. alutacea	HMAS 52742	China	**ON520743**	DQ443438	—	**ON550460**
O. alutacea	HMAS 57844	China	**ON520778**	DQ443439	**ON550447**	**ON550461**
O. apophysata	S-F257062	Germany	KM010077	KM823196	KM823264	KM823389
O. aspera	HMJAU 4166	China	OM743958	OM743964	OM792154	OM792174
O. aspera	HSA 278	China	OM743960	OM743951	OM792153	OM792175
O. aspera	**HSA 251**	**China**	—	**ON520898**	**ON550448**	**ON567204**
O. bomiensis	**HMAS 52743**	**China**	**ON544012**	**ON552240**	**ON550449**	**ON550462**
O. bomiensis	**HMAS 75178**	**China**	**ON550579**	**ON552242**	**ON550450**	**ON550463**
O. borealis	S-F242694	Finland	KM010023	KM823197	KM823265	KM823390
O. brevispora	HMAS 83551	China	OM792189	OM722134	OM792148	OM792168
O. brunneoparva	KH.08.107	Sweden	KM010026	KM823200	KM823268	KM823393
O. brunneoparva	S-F257086	Finland	KM010025	KM823199	KM823267	KM823392
O. bufonia	KH.07.37	Denmark	JN942767	JN941098	KC109262	JN993552
O. bufonia	KH.09.172	Sweden	JN942764	JN941097	KM823272	KM823397
O. bufonia	KH.09.248	Spain	JN942766	JN941084	KM823269	KM823394
O. bufonia	KH.09.249	France	KM010079	KM823201	KM823271	KM823396

O. caeruleopruinosa	H6010805	Finland	KF717575	KM823202	KM823273	KM823398
O. caeruleopruinosa	MT 10082601	Spain	KM010030	KM823203	—	KM823399
O. cantharella	KH.09.125	Sweden	KM010084	KM823205	KM823274	KM823401
O. cantharella	NV 2008.09.16	France	KM010085	KM823204	—	KM823400
O. concinna	KH.09.250	Spain	JN942775	JN941095	KM823276	KM823403
O. concinna	KH.09.183	Sweden	KM010032	JN941089	KM823275	KM823402
O. cupulata	HSA 406	China	OM743959	OM743970	OM792165	OM792177
O. cupulata	HSA 218	China	OM743973	OM743954	OM792166	OM792176
O. daliensis	SEST-06081702	Spain	KM010086	KM823206	KM823277	KM823404
O. filiformis	HMAS 188468	China	OM743949	OM743955	OM792155	OM792178
O. filiformis	BJTC C505	China	MW554241	OM743968	OM792156	OM792179
O. filiformis	BJTC L482	China	MW554466	OM743963	OM792157	OM792180
O. flavidobrunneola	H6010806	Finland	KF717576	KM823209	KM823279	KM823407
O. flavidobrunneola	KH.09.153	Norway	KM010088	KM823207	—	KM823405
O. formicarum	JS.08.63	Sweden	KM010035	KM823212	KM823282	—
O. formicarum	H6003549	Finland	KF717577	KM823211	KM823281	KM823409
O. formicarum	S-F244372	Norway	KM010034	KM823210	KM823280	KM823408
O. gongnaisiensis	**HMAS 69951**	**China**	**ON550580**	**ON552243**	**ON550451**	**ON550464**
O. gongnaisiensis	**HMAS 83574**	**China**	**ON551357**	**ON552244**	**ON550452**	**ON550465**
O. hanseniae	WZ 2202	China	KU987012	KU987024	KU987033	—
O. hanseniae	XF007	China	KU987016	KU987028	KU987035	KU987038
O. hanzhongensis	**610723MF0034**	**China**	KY950470	**ON552257**	**ON550453**	**ON550466**
O. kaushalii	T. Læssøe 6236	Malaysia	KM010119	AF335111	KM823326	KM823455
O. khakicolorata	BJTC FM107	China	OM743967	OM743950	OM792158	OM792181
O. korfii	Z.W. Ge 1913	China	KU987017	KU987029	KU987036	—
O. kunmingensis	HKAS 49452	China	MK850489	—	—	—
O. lactea	HMAS 61359	China	OM721661	DQ443447	—	—
O. leporina	NV 2008.09.28	France	KM010092	KM823214	KM823284	KM823411
O. leporina	OSC 56784	USA	—	KM823215	KM823285	KM823412
O. leporina	H6003548	Finland	KF717578	KM823222	KM823292	KM823421
O. leporina	KH.09.93	Sweden	KM010090	KM823213	KM823283	KM823410
O. minor	KH.98.84	Denmark	KM010041	KM823217	KM823287	KM823414
O. minor	H6008618	Finland	KM010039	KM823219	KM823289	KM823416
O. minor	KH.10.311	Sweden	KM010042	KM823218	KM823288	KM823415
O. mirabilis	KH.01.09	Denmark	JN942769	AY500540	KM823290	KM823419
O. mirabilis	NV 2008.09.14	France	JN942768	JN941094	KM823291	KM823420
O. mirabilis	KH.10.285	Sweden	KM010094	KM823221	—	KM823418
O. nannfeldtii	JS.08.103	Sweden	KM010045	KM823224	KM823294	KM823423
O. nannfeldtii	H6002902	Finland	KF717581	KM823228	KM823297	KM823426
O. nannfeldtii	NV 2008.10.01	France	KM010099	KM823227	KM823296	KM823425
O. nannfeldtii	rh101310	USA	KM010100	KM823226	KM823295	KM823424
O. onotica	OSC 56759	USA	AF072068	JN941088	KM823300	KM823430
O. onotica	C-F-89691	Denmark	JN942773	JN941090	—	KM823427
O. onotica	KH.10.284	Sweden	KP006505	KM823229	KM823299	KM823429
O. onotica	KH.09.132	Norway	KM010103	KC012692	KC109263	JX943828
O. oregonensis	OSC 56745	USA	AF072089	KM823232	KM823303	KM823433
O. oregonensis	Moorefun 58	USA	KM010048	KM823231	KM823302	KM823432
O. oregonensis	Moorefun 31	USA	KM010047	KM823230	KM823301	KM823431
O. papillata	TUR 102134	Finland	KM010105	KM823233	KM823304	KM823434

O. papillata	H6003547	Finland	KF717582	KM823234	KM823305	KM823435
O. parvispora	JS.08.81	Sweden	KM010062	KM823187	KM823255	KM823383
O. parvispora	MCVE 30108	Spain	MK850491	MK850504	—	—
O. parvispora	MCVE 30107	Greece	MK850490	MK850503	—	—
O. parvula	BJTC FM210-A	China	OM743965	OM743966	OM792159	OM792182
O. parvula	BJTC FM210-B	China	OM743962	OM743956	OM792160	OM792183
O. phlebophora	JV06-385	Denmark	KM010049	KM823236	KM823306	KM823436
O. phlebophora	S-F108338	Sweden	KM010050	KM823490	—	—
O. platyspora	KH.09.163	Sweden	KM010106	KM823238	KM823308	KM823438
O. platyspora	JV06-656	Denmark	KM010108	KM823237	KM823307	KM823437
O. plicara	BJTC FM262-A	China	OM743957	OM743971	OM792161	OM792187
O. plicara	BJTC FM262-B	China	OM743961	OM743952	OM792162	OM792186
O. propinquata	NV 2008.09.15	France	KM010111	KM823240	KM823310	KM823440
O. propinquata	KH.09.99	Sweden	KM010109	KM823239	KM823309	KM823439
O. pseudoformicarum	HKAS 101386	China	KY498601	KY498606	—	MG980709
O. pseudoleporina	Moorefun14	USA	KM010113	KM823242	KM823312	KM823442
O. pseudoleporina	rh101910	USA	KM010112	KM823243	KM823313	KM823443
O. purpureobrunnea	BJTC FM1061	China	OM743969	OM743972	OM792163	OM792185
O. purpureobrunnea	BJTC FM1048	China	OM743974	OM743953	OM792164	OM792184
O. purpureogrisea	Z.W. Ge 863 (HKAS	China	KU987011	KU987023	KU987032	KU987037
O. purpureogrisea	WZ 2157 (HMAS72805)	China	KU987015	KU987027	KU987034	OM792170
O. purpureogrisea as O. olivaceobrunnea	HMAS 23948	China	KU987010	KU987022	—	—
O. rainierensis	A.H. Smith 30553 (MICH)	USA	KF717583	KM823245	KM823315	KM823445
O. saliceticola	MCVE 29365	Italy	MG383806	MG383814	—	—
O. shennongjina	**HMAS 53691**	**China**	**ON551356**	**ON552249**	**ON550454**	**ON550467**
O. shennongjina	**HMAS 53692**	**China**	**ON551386**	**ON552248**	**ON550455**	**ON550468**
O. sinensis	HMAS 61360	China	OM722037	DQ443451	—	—
O. sinensis	**HMAS 268390**	**China**	**ON520792**	**ON521126**	**ON550456**	**ON550469**
O. sinensis	**HMAS 268544**	**China**	**ON520802**	**ON521143**	**ON550457**	**ON550470**
O. smithii	OSC 56799	USA	AF072063	JN941087	KM823317	KM823447
O. smithii	ecv3345	USA	JN942771	JN941093	KM823316	KM823446
O. stipitata	HKAS 87865	China	KY498603	KY498608	—	—
O. subformicarum	CMP 1179	Spain	KM010053	KM823246	KM823318	KM823448
O. subformicarum	CL050928-30	Italy	KM010052	KM823247	KM823319	KM823449
O. subformicarum	S-F242696	Spain	KM010054	KM823495	—	—
O. aff. *subformicarum*	FH301035	Mexico	KM010055	KM823249	KM823321	KM823451
O. aff. *subformicarum*	FH301036	Mexico	KM010056	KM823248	KM823320	KM823450
O. subpurpurea	HKAS 54449	China	KU987018	KU987030	OM792150	OM792171
O. subpurpurea	HMAS 97530	China	KU987019	KU987031	—	—
O. subpurpurea as *O. bicolor*	HKAS 54453	China	OM714812	OM722133	OM792152	OM792173
O. subpurpurea as *O. pruinosa*	HKAS 81819	China	KY498602	KY498607	OM792149	OM792169
O. subterranea	RH69	USA	FJ404767	FJ404767	—	—
O. subterranea	RH97	USA	FJ404766	FJ404766	—	—
O. tuomikoskii	NV 2008.09.08	France	JN942777	JN941091	KM823323	KM823453
O. tuomikoskii	KH.09.130	Norway	JN942776	JN941092	KM823322	KM823452
O. tuomikoskii	OSC 56761	USA	AF072085	KM823251	KM823325	KM823454

O. tuomikoskii	H6002901	Finland	KF717585	KM823250	KM823324	—
O. unicisa	ZW Geo65-Clark	USA	KM010118	AY789369	—	—
O. unicisa	KH.06.06	USA	—	KC012693	KC109264	JX943829
O. yunnanensis	HMAS 82166	China	—	DQ443452	—	—
O. sp.	GMFN 2293	Italy	KM010037	KM823476	—	—
O. sp.	MK1081	Sweden	KM010058	KM823501	—	—
O. sp.	MK0942	Sweden	KM010057	KM823500	—	—
O. sp.	KH.09.79	Sweden	KM010120	KM823252	KM823327	KM823456
***O.* sp. 'c'**	**HMAS 85660**	**China**	**ON520805**	**ON521145**	**ON550458**	**ON567203**
***O.* sp**	**HMAS 88262**	**China**	**ON520806**	**ON521147**	**ON550459**	**ON550471**
Warcupia terrestris	CBS 891.69	Canada	MH859473	MH871254	KC109308	JX943832
